# A Delphi Study on the Management of Female Infertility by Homeopaths in South Africa

**DOI:** 10.4102/hsag.v29i0.2771

**Published:** 2024-11-08

**Authors:** Robyn Anderson, Janice Pellow, Tebogo Tsele-Tebakang, Elizabeth Solomon

**Affiliations:** 1 Department of Complementary Medicine, Faculty of Health Sciences, University of Johannesburg, Johannesburg, South Africa

**Keywords:** female infertility, Delphi technique, homeopathy, supplements, diet, lifestyle

## Abstract

**Background:**

Infertility affects millions of people worldwide and has a negative social and psychological impact on the lives of infertile couples. Homeopathy offers an alternative treatment option for female infertility; however, there is currently no research describing how homeopaths manage this condition in their practice.

**Aim:**

To determine homeopathic practitioners’ approaches to managing female infertility.

**Method:**

The electronic Delphi technique consisting of three rounds was used to establish consensus among homeopaths. Twelve registered homeopaths with a minimum of 5 years of clinical experience in managing female infertility participated, 11 of whom completed the study. In round one, participants elaborated on interventions found beneficial in clinical practice for female infertility. Responses were qualitatively analysed to create a structured list of items; participants rated their level of agreement with these items on a 5-point Likert scale in the second and third rounds. Consensus was determined for items that reached an agreement level of 75% or more.

**Results:**

Thirty-two statements achieved expert consensus, which were placed into the following categories: homeopathic treatment, dietary and lifestyle modifications, supplementation and referrals.

**Conclusion:**

The results of this study provide a baseline depicting the homeopathic approach to treating female infertility. Several research gaps have been identified and further studies are necessary to explore these interventions to improve future patient care.

**Contribution:**

This study highlights the various approaches used by homeopathic practitioners in the management of female infertility.

## Introduction

Infertility is defined as the failure to establish a clinical pregnancy after 12 months of regular, unprotected sexual intercourse, and is estimated to affect millions of people worldwide (Vander Borght & Wyns 2018). A systematic review and meta-analysis conducted by Cox et al. (2022) explored the prevalence of infertility as reported in studies published between 1990 and 2021, which showed that estimates for 1-year and lifetime prevalence were 12.6% and 17.5%, respectively. Approximately 37% of couples who are unable to conceive experienced factors related to female infertility, with ovulatory disorders, pelvic adhesions, tubal obstruction, other tubal or uterine abnormalities and hyperprolactinaemia identified as underlying causes (Walker & Tobler 2022). Other factors that can negatively impact female fertility include primary ovarian insufficiency, polycystic ovarian syndrome (PCOS), uterine fibroids, endometriosis and endometrial polyps, increasing age, obesity, extreme energy restriction, excessive exercise, tobacco and alcohol intake, and stress (Vander Borght & Wyns 2018).

Homeopathy is a holistic healing method that provides an individualised treatment approach to restore health (Viganò, Nannei & Bellavite 2015). Research related to the effectiveness of homeopathy in treating female infertility is limited; however, available studies have suggested a potential improvement in fertility parameters using homeopathic treatment (Lobo, D’Cunha & Lobo 2018; RajachandraSekar et al. 2022). According to their scope of practice, homeopathic practitioners in South Africa can diagnose, treat and prevent any illness or deficiency using homeopathic remedies, dietary advice and health supplementation (AHPCSA 2019). There has been no study done to date to determine homeopathic practitioners’ approaches to the management of female infertility.

### Aim

The study aimed to determine homeopathic practitioners’ approaches to managing female infertility.

### Objectives

The study objectives were the following:

- To investigate, using the Delphi technique, the practices of homeopathic practitioners in the management of female infertility.
- To determine expert consensus from homeopathic practitioners on the methods used to manage female infertility.

## Research methods and design

### Study design

A three-round, electronic Delphi technique was used. The Delphi is a structured iterative method that provides a group consensus strategy by including expertise from several individuals to answer a complex research question (Niederberger & Spranger 2020). After conducting a thorough literature search on the research problem, a semi-structured questionnaire was developed for round one. Before commencing the study, it was determined that a minimum of two rounds would be conducted. The first round consisted of open-ended and closed-ended questions to gather participant demographics and the interventions they used for treating female infertility. After analysing these responses, a second-round questionnaire was developed, where participants rated the importance of the interventions on a Likert-type scale. In the third round, a refined set of items was presented for participants to rate their level of agreement. The process was concluded after three rounds, once a consensus of 75% agreement was reached.

### Population and sampling strategy

Homeopathic practitioners registered with the Allied Health Professions Council of South Africa (AHPCSA), with a minimum of 5 years of experience treating female infertility, were purposively recruited to ensure eligibility. There are currently 582 homeopathic practitioners registered in South Africa (Allied Health Professions Council of South Africa n.d.). Furthermore, participants were identified through leads obtained from the Homeopathic Association of South Africa (HSA) and snowball sampling. Participants were recruited via an emailed invitation detailing the study. While sample size may vary across Delphi studies, the average number of participants included is usually in the low to medium double-digit range (Niederberger & Spranger 2020).

### Data collection

In each Delphi round, electronic surveys were used to collect data via a dedicated online platform. Participants were requested to give consent before the completion of each round, and each questionnaire took approximately 15 min to complete. Participants were given 14 days to complete the survey and were sent weekly reminder emails. The first-round questionnaire consisted of both open- and closed-ended questions, pertaining to: (1) demographics (age, gender, qualification/s, years in practice, average number of infertility patients seen per year) and (2) the interventions used, which included homeopathic treatment, dietary and lifestyle advice, and supplementation. Findings from round one were qualitatively analysed using the content analysis method (Erlingsson & Brysiewicz 2017). The second round allowed participants to rate the importance of the items identified in round one using a Likert-type scale and to further elaborate on the responses given. The ratings of this scale were ‘not recommended’, ‘unimportant’, ‘somewhat important’, ‘very important’ or ‘essential’. In the third round, participants rated their level of agreement with the refined series of items as ‘strongly disagree’, ‘disagree’, ‘unsure’, ‘agree’ or ‘strongly agree’. The level of agreement of individual statements was analysed for central tendency (median), percentage of agreement (%) and variance (interquartile range [IQR]). The defined level of agreement in Delphi studies varies in the literature; however, a threshold of 60% or higher is identified in most cases (Niederberger & Spranger 2020). For this study, consensus was defined as a combined agreement level (agree or strongly agree) of 75% or more among participants.

### Ethical considerations

Participation in this study was entirely voluntary, with consent being required for each round.

Ethical approval to conduct this study was obtained from the University of Johannesburg’s Faculty of Health Sciences Research Ethics Committee with the following ethical clearance number: REC-1094-2021. Participants were informed of the details of the study through an information sheet that was sent to them when they were invited to join the study. The researcher was available to answer any questions the participants had. If participants wished to withdraw from the study, they were free to do so at any time. Each participant was assigned a unique code, recorded in a spreadsheet listing each code with an email address, ensuring anonymity throughout. The data linking the participant to the code, as well as any completed questionnaires, were kept on a password-protected computer that only the researcher had access to and will be kept for 5 years, ensuring confidentiality. No identifying data was published. The questionnaire was conducted electronically and there were no hard copies available. There were no anticipated risks in this study. Participants were given access to the results on request.

## Results

The Delphi study was conducted in three rounds over 14 months, between July 2021 and September 2022. Fifty homeopaths were invited to participate; 12 completed rounds one and two, and 11 completed round three (91.7% response rate). Table 1 represents the demographic and background information of the participants. Most were female (*n* = 10; 83.3%) between the ages of 45 and 54 years (*n* = 6; 50%). Participants were asked to estimate the average number of female infertility patients seen per year; the results showed that female infertility is a commonly treated condition, with 41.7% (*n* = 5) of participants treating 41 or more patients per year.

**TABLE 1:** Demographic and background information of participants.

Characteristics	*n*	%
**Homeopathic qualification**		
Master of Technologiae in Homeopathy	12	100.0
**Additional qualifications**		
Diploma in Acupuncture	1	8.3
Bachelor of Medicine and Bachelor of Surgery	1	8.3
Applying Functional Medicine in Clinical Practice	1	8.3
Master of Technologiae in Chiropractic	1	8.3
Bachelor of Science	1	8.3
**Gender**		
Male	2	16.7
Female	10	83.3
**Age (years)**		
25–34	2	16.7
35–44	3	25.0
45–54	6	50.0
55–64	1	8.3
**Number of years in practice**		
5–9	1	8.3
10–15	2	16.7
16–20	4	33.3
21–25	3	25.0
26–30	2	16.7
> 31	0	0.0
**Estimated number of female infertility patients seen per year**		
1–10	2	16.7
11–20	4	33.3
21–30	1	8.3
31–40	0	0.0
41 or more	5	41.7

Participants were asked to describe their homeopathic approach to the management of female infertility and the type of interventions used. The responses from round one were arranged into themes based on categories of homeopathic treatment, dietary and lifestyle modifications, health supplements and referrals. In the second round, participants rated the importance of this list of items, and they also had the opportunity to provide further responses on specific interventions used. These data were added to the questionnaire for the third round. Participants once again rated their level of agreement. Of the 61 individual statements presented in round three, 32 achieved a rater agreement consensus of 75% or more (Table 2). The remaining 29 statements did not reach consensus in rounds two and three and were excluded from the final list of statements (Table 3).

**TABLE 2:** Statements from round three achieving consensus.

When treating female infertility, it is important to …	Level of Agreement (*N* = 11)		Median	IQR
	%	*n*		
**Homeopathic treatment**				
Perform a thorough history taking	100.0	11	5	0
Repertorise	81.8	9	4	1
Schedule regular follow-ups	100.0	11	5	1
Treat layer by layer	81.8	9	4	1
Use nosodes	90.9	10	4	1
Use homeopathic mother tinctures	90.9	10	4	1
Use gemmotherapy	90.9	10	4	1
Use organotherapy	90.9	10	4	1
**Dietary and lifestyle modifications**				
Increase fruit and vegetable intake	100.0	11	5	1
Eliminate/reduce wheat and gluten	81.8	9	5	1
Eliminate/reduce sugar and refined carbohydrates	90.9	10	5	1
Eliminate/reduce dairy	81.8	9	5	1
Eat frequently throughout the day	81.8	9	4	1
Eliminate/reduce processed foods	100	11	5	1
Eliminate/reduce canola oil	90.9	10	5	1
Consume a high fibre diet	90.9	10	4	1
Ensure adequate water intake	100.0	11	5	0
Eliminate alcohol	81.8	9	5	1
Eliminate/reduce caffeine	90.9	10	4	1
Eliminate carbonated drinks	81.8	9	5	1
Eliminate cigarette smoking	90.9	10	5	0
Ensure adequate sleep	100.0	11	5	0
Manage stress	100.0	11	5	0
**Supplementation**				
Recommend a magnesium supplement	81.8	9	4	1
Recommend supplements for liver support	81.8	9	4	1
Recommend supplements for adrenal support	100.0	11	4	1
**Referrals**				
Refer to a gynaecologist	90.9	10	5	1
Refer to an endocrinologist	81.8	9	4	0
Refer for acupuncture	90.9	10	4	1
Refer for an ultrasound	90.9	10	5	1
Refer for reflexology	81.8	9	4	0
Refer for psychological care	90.9	10	4	1

IQR, interquartile range.

**TABLE 3:** Items excluded after round three (*N* = 11).

Statement	Level of agreement %	Median	IQR
**Homeopathic treatment**			
Use a single homeopathic remedy	72.7	4	3
Use homeopathic complexes	72.7	4	2
Use biopuncture	54.5	4	2
Use organ drainage	63.6	4	2
**Dietary and lifestyle advice**			
Minimise soy products	72.7	4	2
Eliminate/reduce red meat	45.5	3	2
Eliminate/reduce vinegar	27.3	3	1
Recommend using fluoride-free toothpaste	54.5	4	1
**Supplementation**			
Recommend a multivitamin	63.6	4	2
Recommend a vitamin B complex supplement	72.7	4	1
Recommend an iodine supplement	27.3	3	1
Recommend a zinc supplement	63.6	4	1
Recommend coenzyme Q10/ubiquinol	54.5	4	1
Recommend N-acetyl cysteine	36.4	3	1
Recommend a selenium supplement	72.7	4	1
Recommend an omega-3 supplement	72.7	4	2
Recommend a cinnamon supplement for PCOS	36.4	3	1
Recommend a berberine supplement for PCOS	63.6	4	2
Recommend a chromium supplement for PCOS	54.5	4	1
Recommend inositol for PCOS	63.7	4	2
Recommend diindolylmethane (DIM) for oestrogen dominance	72.7	5	2
Recommend omega-6 for oestrogen dominance	27.3	2	2
**Referrals**			
Refer for IVF or ICSI	36.4	3	2
Refer to a dietitian/nutritionist	45.5	3	2
Refer to a chiropractor	27.3	3	2
Refer for osteopathy	36.4	3	2
Refer for craniosacral therapy	54.5	4	1
Refer to traditional Chinese medicine (TCM)	63.7	4	2
Refer to DNA analysis	72.7	4	2

IQR, interquartile range; PCOS, polycystic ovarian syndrome; IVF, in vitro fertilization; ICSI, intracytoplasmic sperm injection.

## Discussion

In this study, 11 participants completed a three-round electronic Delphi survey to establish homeopathic approaches to managing female infertility in the South African setting. Consensus was reached on 32 statements, comprising 8 items related to homeopathic treatment, 15 items related to dietary and lifestyle advice, 3 items on supplements and 6 items to referrals. Participants agreed on the importance of performing thorough history taking and scheduling regular follow-ups. Medical history taking is considered an essential tool in reaching a diagnosis and providing optimal care (Nichol, Sundjaja & Nelson 2022), while follow-up consultations are an important element of good practice that allows the practitioner to monitor treatment outcomes and disease progression (Brand & Stiggelbout 2013).

Regarding homeopathic treatment, 81.8% of participants agreed on the importance of repertorisation and treating the patient in a layered approach. Repertorisation is a process where symptoms and modalities elicited from history taking are classified into rubrics, using a homeopathic repertory, and then graded to select the most appropriate homeopathic remedy for a specific case. Repertorisation, whether conducted manually or using software, is a useful tool that makes searching for homeopathic intervention faster and more accurate (Gray, Pracjek & Straiges 2023). The approach of addressing a case layer by layer is based on the belief that patients have separate layers of disease, and each layer requires its own homeopathic prescription. These layers include the miasmatic, constitutional, fundamental and lesion layers. The miasmatic layer refers to hereditary predispositions that affect an individual; the constitutional layer focusses on the typology of an individual; the fundamental layer involves the traits a person acquires throughout their lives that later affect their constitution, while the lesion layer relates to pathological symptoms associated with a disease process (Vithoulkas & Chabanov 2023). There is a scarcity of research available on the homeopathic approach of treating layer by layer and this identifies an interesting research niche that needs further exploration. Consensus could not be reached among homeopaths on prescribing a single remedy, which aligns with the classical homeopathic approach, or complex remedy, which relates to a more clinical approach. Both treatment approaches achieved similar consensus, at 72.7% of the participants. Therefore, homeopaths varied in their preferences and prescribing style and thus preferred to tailor their treatment plan to the individual.

Additionally, 90% of participants mention using nosodes, organotherapy, gemmotherapy and mother tinctures in their treatment approach. Nosodes are homeopathic remedies obtained from the potentisation (serial dilution and succussion) derived from an element of a disease and are administered to prevent or treat disease (Rieder & Robinson 2015), while organotherapy (sarcodes) are potentised remedies prepared from healthy secretions, tissues or organs that are administered to promote optimal organ function (Bioregulatory Medicine Institute, n.d). However, there is scarce research available on the usefulness of nosodes or organotherapy in the treatment of female infertility.

Gemmotherapy is a branch of phytotherapy where plant buds and shoots are macerated in water, alcohol and glycerol to produce remedies in a D1 (1:10 dilution) potency (Di Vito et al. 2020). Mother tinctures, on the other hand, are liquid preparations of fresh plant material mixed with alcohol or water, usually in a 1:10 dilution, prepared according to the methods laid out in the homeopathic pharmacopoeia (Scheepmaker & Gower 2011). Participants named various individual gemmotherapy and mother tinctures they used to treat possible underlying issues related to infertility, such as female hormonal disorders, and thyroid disorders and to provide liver and adrenal support. Of those mentioned, Vitex agnus-castus mother tincture was reported as being a useful remedy for female infertility by 91.7% of participants. Vitex agnus-castus, also known as chaste tree, has been shown to regulate hormonal imbalances and may be useful in the treatment of PCOS, premature ovarian failure, hypothalamic dysfunction, hyperprolactinaemia, endometriosis, premenstrual syndrome (PMS) and mastalgia (Akbaribazm, Goodarzi & Rahimi 2021). However, there are currently no clinical studies on its use as a mother tincture preparation to treat female infertility.

The use of specific remedies was reported in round two; however, consensus was not reached for any individual intervention. This is not surprising considering homeopathic prescribing is based on the individuals’ presentation (Viganò et al. 2015). Research has shown that diet is important in female fertility (Panth et al. 2018). Participants in this study agreed that increasing fruit, vegetables, fibre and water intake was essential to promote fertility. Literature highlights a diet of vegetables, whole grains, unsaturated fats and fish is associated with improved fertility outcomes (Panth et al. 2018; Skoracka et al. 2021).

Most participants agreed that ultra-processed foods, gluten, sugar and refined carbohydrates, carbonated drinks, dairy products, alcohol and caffeine should be reduced or eliminated from the diet to enhance fertility. These findings are supported by Pagliai et al. (2021), Panth et al. (2018) and Skoracka et al. (2021). It is suggested that reducing sugar and refined carbohydrate intake may improve fertility rates in certain women by decreasing insulin levels, promoting ovulation and improving hormonal balance (McGrice & Porter 2017). Research on dairy consumption and fertility is ambiguous, with some studies suggesting a positive effect on fertility and ovulation, while others show potential risks, particularly with low-fat dairy. More research is needed to confirm whether avoiding dairy is beneficial for fertility (Janiszewska, Ostrowska & Szostak-Węgierek 2020).

Most participants (90.9%) in this study also recommended eliminating canola oil from the diet; however, this opinion differs from the literature. The benefits and safety of canola oil have been widely studied with mixed results; however, most studies show favourable benefits associated with its consumption. One such study showed the positive effects of canola oil on the lipid profile and liver function in women with PCOS (Yahay et al. 2021).

Also, in contrast to available literature, around 81% of participants recommended avoiding long periods of fasting between meals. Evidence shows that periods of fasting, the frequency and specific time of meals consumed within a day, produce various physiological effects. For instance, following a regular eating pattern where most energy is consumed early in the day, eating only 2–3 meals a day and undertaking limited periods of regular fasting may all provide positive health benefits (Paoli et al. 2019). Concerning fertility, intermittent fasting has been shown to reduce androgen levels and raise sex hormone-binding globulin (SHBG) levels in obese women, as well as reduce hyperandrogenism, and improve menstruation rates and fertility in females with PCOS (Cienfuegos et al. 2022). Intermittent fasting may therefore be beneficial to some patients, depending on the underlying cause of their infertility.

The lifestyle modifications that achieved consensus in this study included eliminating cigarette smoking, getting adequate sleep and stress management. Even though smoking is associated with an increased incidence of female infertility, there are inconsistencies in the literature concerning the quantity, length of time, and recency and their effects on fecundability. It is found that women who actively smoke at high intensity and for a long duration tend to have poorer fertility outcomes (Wesselink et al. 2019). The release of hormones needed for the proper reproductive function of humans is closely linked to the body’s natural circadian rhythms. Sleep deprivation is reported to be associated with infertility in females; this is because of an interference of the hypothalamic–pituitary–adrenal (HPA) axis and irregular production of female sex hormones. Sleep deprivation in women has also been found to be associated with failed embryo implantation, anovulation and amenorrhea (Lateef & Akintubosun 2020). All participants agreed that managing stress was essential in the treatment of infertility. Prolonged stress produces neuroendocrine disturbances which negatively impact fertility. Infertility causes anxiety for many women, and multiple studies have shown the benefit of psychological interventions to manage stress while undergoing infertility treatment (Palomba et al. 2018; Rooney & Domar 2018).

Participants recommended various health supplements in the management of female infertility. The specific supplements that achieved consensus in this study included magnesium and liver and adrenal gland support. Supplementation with magnesium has been shown to improve insulin resistance and glucose metabolism and thus may assist fertility parameters in women with PCOS and metabolic disorders (Porri et al. 2021; Skoracka et al. 2021). Magnesium also plays a role in the stress response and studies show that psychological stress could result in a magnesium deficiency; conversely, magnesium deficiency could increase one’s susceptibility to stress (Pickering et al. 2020). Supplements for liver support were seen as important by 81.8% of participants. The liver modulates the metabolism of sex hormones and transport to tissues via SHBG (Grossmann et al. 2019), and chronic liver disease may affect the ability to conceive (Rahim et al. 2021). In addition, certain conventional medications such as clomiphene used to treat female infertility may cause ovarian hyperstimulation syndrome, which is associated with elevated levels of liver enzymes (LiverTox 2017), indicating the possible benefit of liver support. The following supplements did not achieve consensus, but were recommended by a large portion (72.7%) of the participants: a vitamin B complex, selenium, omega-3 and diindolylmethane (DIM).

The adrenal glands play an essential role in the body’s stress response and secrete various hormones involved in immunity, metabolism, and salt and water balance (Megha et al. 2022). Adrenal dysfunction is associated with premature ovarian insufficiency and decreased fertility in females (Bensing, Giordano & Falorni 2020). All participants in this study reported recommending supplements for adrenal support.

Statements related to referrals were also obtained. Referral to a gynaecologist, an endocrinologist and for ultrasound can be seen as good practice in cases of female infertility. Complementary medicine options referred for included acupuncture (90.9%) and reflexology (81.8%). Acupuncture has been described as a beneficial treatment for female infertility that assists in improving ovarian function and hormonal imbalances (Quan et al. 2022); however, there is scarce evidence relating to reflexology and female fertility (Holt et al. 2009). Ten out of 11 participants agreed with referring patients to a psychologist. Psychological stress impairs fertility by increasing oxidative stress in the body, thus impairing oocyte quality and ovarian function (Pandey et al. 2028). In addition, infertility is associated with devastating psychological and emotional consequences. Studies have shown that participating in group psychological therapy improves the mental health, fertility stress and pregnancy rates of women undergoing fertility treatment (Warne, Oxlad & Best 2023).

### Strengths and limitations

This study had several strengths. The participants were made up of homeopaths with varying clinical experiences, five of whom held other qualifications within the health field. The Delphi technique is useful in fields with limited research such as homeopathy, as it combines the knowledge of multiple practitioners who are considered knowledgeable and generates insights into the possible intervention. Using electronic questionnaires allowed participants to complete the questionnaire at their convenience and contributed to the low attrition rate (0.83%). The limitation of the study include the small sample size and the difficulty in obtaining consensus due to the inherently individualised nature of homeopathic treatment.

## Conclusion

Infertility is a condition for which females may consider visiting a homeopath for intervention. A noteworthy point is that homeopathic practitioners adopt an individualised approach to the management of infertility. Each participant had their preferences for specific homeopathic remedies, dietary and lifestyle changes, and health supplements, which were based on their own experiences in practice. The results of this study may form the basis for future clinical studies in this field and help identify research gaps. There is a great need to conduct studies to verify these findings and provide evidence to inform best practice protocols.
